# Fluorine-18: Radiochemistry and Target-Specific PET Molecular Probes Design

**DOI:** 10.3389/fchem.2022.884517

**Published:** 2022-06-29

**Authors:** Yunze Wang, Qingyu Lin, Hongcheng Shi, Dengfeng Cheng

**Affiliations:** ^1^ Department of Nuclear Medicine, Zhongshan Hospital, Fudan University, Shanghai, China; ^2^ Institute of Nuclear Medicine, Fudan University, Shanghai, China; ^3^ Shanghai Institute of Medical Imaging, Shanghai, China

**Keywords:** fluorination, fluoroalkylation, 18 F-radiolabeling, PET radiotracers, PET imaging

## Abstract

The positron emission tomography (PET) molecular imaging technology has gained universal value as a critical tool for assessing biological and biochemical processes in living subjects. The favorable chemical, physical, and nuclear characteristics of fluorine-18 (97% β^+^ decay, 109.8 min half-life, 635 keV positron energy) make it an attractive nuclide for labeling and molecular imaging. It stands that 2-[^18^F]fluoro-2-deoxy-D-glucose ([^18^F]FDG) is the most popular PET tracer. Besides that, a significantly abundant proportion of PET probes in clinical use or under development contain a fluorine or fluoroalkyl substituent group. For the reasons given above, ^18^F-labeled radiotracer design has become a hot topic in radiochemistry and radiopharmaceutics. Over the past decades, we have witnessed a rapid growth in ^18^F-labeling methods owing to the development of new reagents and catalysts. This review aims to provide an overview of strategies in radiosynthesis of [^18^F]fluorine-containing moieties with nucleophilic [^18^F]fluorides since 2015.

## Introduction

**FIGURE 1:**
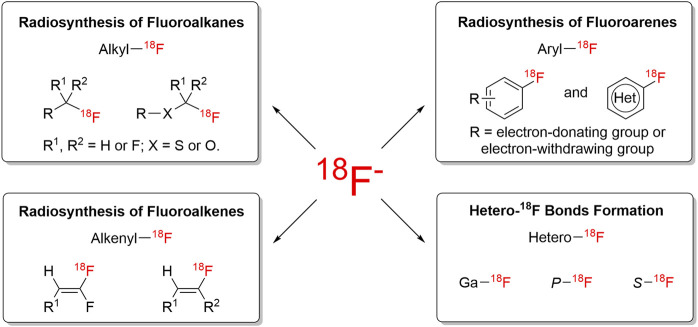
Overview of ^18^F-chemistry.

Positron emission tomography (PET) is a non-invasive and quantitative imaging technology for assessing biological processes *in vivo* (Preshlock et al., 2016). Due to the high sensitivity of PET, the concentration of radiolabeled probes required was as few as the picomolar scale (10^−6^–10^−8^ g). Therefore, the mass effect is not to be highly considered in probe design and labeling experiments. Compared with alternative positron-emitting radioisotopes (e.g., ^11^C, ^13^N, ^15^O, ^68^Ga, ^89^Zr), ^18^F has distinct physical advantages, including 1) simple decay profile (97% positron emission and 3% electron capture), 2) lower positron energy (maximal positron energy of 0.635 MeV) resulting in short positron range and high resolution, 3) favorable physical half-life (109.8 min half-life) suitable for labeling and *in vivo* evaluation, and 4) flexible for labeling viable molecules by different labeling strategies. Based on these unique and advantageous characteristics of fluorine-18, ^18^F-labeled radiotracers have become a hot topic in molecular probe design. Whereas challenges still exist, considering fast labeling and favorable radiochemical yields have to be given higher priority in clinical practice. In recent years, many efforts have been made to develop new methods and new reagents for radiosynthesis of [^18^F]fluorine-containing moieties. Radiofluorination and radiofluoroalkylation reactions have been excellently reviewed by Gouverneur and co-workers (Preshlock et al., 2016), and Vugts and co-workers (Born et al., 2017) from 2010. Herein, this review focused on summarizing the recent developments in ^18^F-labeling methods and application in PET tracer design since 2015, according to the structures of desired radiolabeled complexes in each case, the following characteristics will be discussed: 1) radiosynthesis of fluoroalkanes with [^18^F]fluorides; 2) radiosynthesis of fluoroarenes with [^18^F]fluorides; 3) radiosynthesis of fluoroalkenes with [^18^F]fluorides; 4) heteroatom-^18^F bonds formation (Figure 1). Unless otherwise mentioned, radiochemical yield (RCY) and radiochemical conversion (RCC) are calculated without time-decay; R means both electron-donating groups and electron-withdrawing groups are capable of this reaction.

## Radiosynthesis of Fluoroalkanes With [^18^F]Fluorides

**FIGURE 2:**
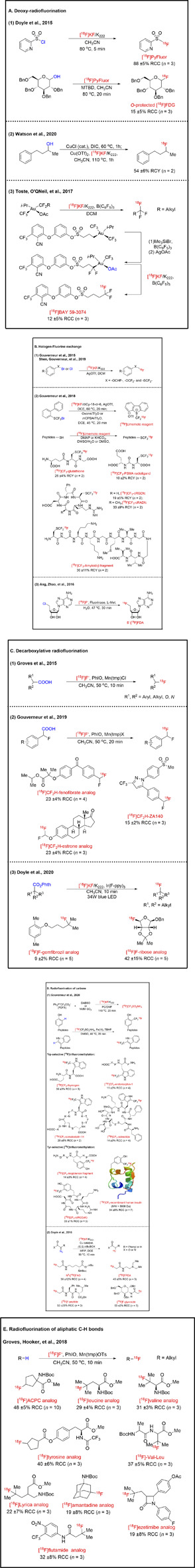
(Continued).

Nucleophilic [^18^F]fluorination has been commonly used to generate aliphatic C-^18^F bonds (Deng et al., 2019). However, labeling precursors with alcohol-derived leaving groups or halides are easily decomposed or eliminated to the corresponded alkenes under harsh conditions (high temperatures and basicity) (D'Angelo and Taylor, 2016; Cai et al., 2008). To resolve this issue, nucleophilic [^18^F]fluorination of aliphatic alcohol and halides under mild conditions remained to be discussed. Other novel labeling precursors, such as carboxylic acid, and carbene precursors will be also discussed (Figure 2).

Aliphatic C−^18^F bonds are constructed by nucleophilic substitution of alcohol-derived leaving groups with [^18^F]fluorides in the presence of phase transfer reagents. The transformation of alcohols into corresponding ^18^F-labeled alkyl compounds typically involves two steps, the formation of alcohol-derived leaving groups was firstly required, including mesylate, triflate, tosylate, and followed by nucleophilic [^18^F]fluorination (Deng et al., 2019). Alcohols are frequently-used moiety in natural products and pharmaceutical molecules. Herein, the research on direct deoxy-radiofluorination benefits PET tracer designing. Previously, deoxy-radiofluorination required a^19^F carrier to generate adequate electrophile *in situ* to react with the tiny amount of ^18^F anion (Jelinski, et al., 2001). Doyle and co-workers reported the first example of a no-carrier-added deoxy-radiofluorination which applied direct conversion of alcohols into alkyl fluorides in one pot (Nielsen et al., 2015) (Figure 2A1). As proof of concept, the [^18^F]PyFluor was achieved in 88% of radiochemical conversion (RCC) by reacting 2-pyridinesulfonyl chloride with [^18^F]KF/K_222_ at 80°C for 5 min. Using this reagent, hydroxy protected [^18^F]FDG was achieved by deoxy-radiofluorination in 15% of RCC at 80°C for 20 min. This radiolabeling protocol shows particularly useful with substrates for which the sulfonate ester cannot be isolated. After that, Watson and co-workers developed an efficient method for deoxy-radiofluorination of alcohols using [^18^F]CuF_2_ (generated by Cu(OTf)_2_ and [^18^F]KF *in situ*) in 54% of radiochemical yield (RCY) (Sood et al., 2020) (Figure 2A2). In addition, alcohols were active by *N,N*′-diisopropylcarbodiimide (DIC), and CuF_2_. Elimination byproducts are often generated when radiofluorination of sulfonate-activated secondary alcohols (Cai et al., 2008). They obtained the pure ^18^F-labeled product directly from the alcohol substrate without the elimination of byproducts. Toste, O'QNeil, and co-workers developed a formal deoxy-radiofluorination for radiosynthesis of [^18^F]trifluoromethyl moiety (Levin et al., 2017) (Figure 2A3). The unique biological properties of the trifluoromethyl group have led to their ubiquity in pharmaceutical complexes (Tsui and Hu, 2019). They showed the borane catalyzed formal C (sp^3^)-CF_3_ reductive elimination from an Au(III) complex resulted in the formation of [^18^F]alkyl-CF_3_ compounds. Radiofluorination of the Au(III)-OAc complexes, fluorine-18 substitutes acetate group, are formal deoxy-radiofluorination reactions. The Au(III) complexes (labeling precursors) were prepared by migration-insertion of the alkyl fragment in presence of borane and bromotrimethylsilane (TMSBr) and then *via* anion change with AgOAc. They considered the Au(III)-OAc complexes to exhibit an appropriate blend of stability and reactivity to enable nucleophilic reductive elimination. ^18^F-labeled Bayer lead compound BAY 59–3,074, a cannabinoid agonist (Vry et al., 2004), was radio-synthesized in 12% of RCC with the molar activity of 0.3 GBq/μmol. This protocol provided an important proof of concept in the radiosynthesis of [^18^F]trifluoromethyl groups by a retrosynthetic paradigm involving C-F reductive elimination.

Except for the above-mentioned alcohol-derived leaving groups, aliphatic halides are also capable of nucleophilic [^18^F]fluorination, while showing lower reactivity (Deng et al., 2019). The methods to introduce [^18^F]fluorine anion into organic compounds via halogen-fluorine exchange rely on thermal activation only and limit to aryl-CF_2_X substrates (Suehiro et al., 2011; Martinez et al., 2012). Gouverneur and co-workers developed a novel strategy which for the first-time gave access to a range of respective transformations of aryl-OCHFCl, -OCF_2_Br and -SCF_2_Br to aryl-[^18^F]OCHF_2_, -[^18^F]OCF_3_ and -[^18^F]SCF_3_ derivatives with silver(I) [^18^F]fluoride (generated by halogenophilic silver(I) triflate and [^18^F]fluoride) (Khotavivattana et al., 2015) (Figure 2B1). The molar activities range from 0.04 GB GBq/μmol to 0.17 GBq/μmol. Among this research, they indicated the order of reactivity towards silver [^18^F]fluoride: aryl-OCHFCl > aryl-CF_2_Br ≈ aryl-CHFCl > aryl-SCF_2_Br > aryl-OCF_2_Br. Shen, Gouverneur, and co-workers prepared [^18^F]ArylSCF_3_ compounds in a similar way (Wu et al., 2019).

[^18^F]5-(trifluoromethyl)dibenzothiophenium trifluoromethanesulfonate ([^18^F]Umemoto reagent) has been prepared from [^18^F]fluoride by Gouverneur and co-workers *via* bromine-fluorine exchange and cyclization with the molar activity of 0.08 GBq/μmol (Verhoog et al., 2018) (Figure 2B2). Compared with previous [^18^F]trifluoromethylation methods, this method provided a direct way for [^18^F]trifluoromethylation of unmodified peptides at the thiol cysteine residue with high chemoselectivity. Function groups such as asparagine, glutamine, methionine, glutamic acid, proline, threonine, serine, tyrosine, lysine, or arginine were compatible for this [^18^F]trifluoromethylation with RCCs superior to 55%. Glutathione and ((1-carboxy-2-mercaptoethyl)-carbamoyl)-glutamic acid, a core structure found in PET radioligands targeting prostate-specific membrane antigen (PSMA) (Schwarzenboeck et al., 2017), underwent successful thiol [^18^F]trifluoromethylation respectively in 26 % and 10% of RCY. Radiolabeled Arg-Gly-Asp (RGD) peptides have been a focus for noninvasive assessment of angiogenesis because of their high affinity and selectivity for integrin αvβ_3_ (Hatley et al., 2018). The [^18^F]trifluoromethylation was performed with a cyclic peptide containing the RGD sequence and the radiolabeled cRGDfC and cRADfC were purified and isolated by prep-HPLC in 19% and 33% of RCY. Biodistribution studies by imaging and dissection show that [^18^F]CF_3_-cRGDfC is predominantly excreted by the hepatobiliary route and to a lesser extent by the kidneys. The absence of uptake in the bones indicated that [^18^F]CF_3_-cRGDfC is metabolically stable toward [^18^F]SCF_3_ elimination and that no [^18^F]F^−^was released (Gadais et al., 2017). Beta-amyloid peptide fragments also underwent successful [^18^F]trifluoromethylation in 30% of RCY.

The discovery and characterization of naturally occurring fluorinase enzymes provide an opportunity to synthesize organofluorine compounds by utilizing biocatalysts (O'Hagan and Deng, 2015). O’Hagan et al. (2002) demonstrated the application of the fluorinase enzymes in the fluorination for the first time, then in a following work, O’Hagan and co-workers radio-synthesized 5'-[^18^F]FDA in presence of fluorinase FlA (Martarello et al., 2003). Ang, Zhao, and co-workers employed the evolved variants, fah 2081 (A279Y) and fah2114 (F213Y, A279L), to smoothly radiosynthesize 5'-[^18^F]FDA, with overall radiochemical conversion more than 3-fold higher than FlA1 (Sun et al., 2016) (Figure 2B3).

During the past decades, remarkable advances have been made in the area of decarboxylative fluorination. However, these recently developed methods are mainly based on electrophilic fluorination reagents, the research on decarboxylative fluorination with nucleophilic fluorination reagents is rare (Liang et al., 2013; Brooks et al., 2014). Groves and co-workers reported manganese catalyzed decarboxylative radiofluorination and the RCCs ranged from 20 % to 50% (Huang et al., 2015) (Figure 2C1). Compared to present decarboxylative fluorination methods which are based on electrophilic fluorination reagents, the major advantage of this fluoride-based decarboxylative fluorination reaction is its applicability to ^18^F-labeling with [^18^F]fluoride. Based on their previous work, they considered the tedious azeotropic [^18^F]KF drying step would be eliminated by directly eluting [^18^F]fluoride from the ion exchange cartridge with a solution of the [Mn (salen)]OTs catalyst. Gouverneur and co-workers reported radiosynthesis of [^18^F]ArCF_2_H via Mn mediated decarboxyl-radiofluorination with [^18^F]fluoride (Sap et al., 2019) (Figure 2C2). This reaction is tolerated with various functional groups, such as ether, alkyl, aldehyde, ketone, pyridine, triazole, pyrazole, and dibenzofuran motifs. The higher RCCs were obtained for electron-rich arenes. Fenofibrate analog, COX-II inhibitor ZA140, and estrone analog were also successfully radiolabeled. Doyle and co-workers developed another redox-neutral decarboxylative radiofluorination of *N*-hydroxyphthalimide esters with [^18^F]KF under photoredox catalytic conditions with the typical molar activity of 36.6 GBq/μmol (Webb et al., 2020) (Figure 2C3). To stable the carbocation intermediate, the reacting carbon atom bearing bi-alkyl or aryl is necessary. A common limitation in nucleophilic fluorination methods in delivering secondary benzylic fluorides elimination to styrene byproducts (Sladojevich et al., 2013). However, less than 5% elimination products were observed in the fluorination of the secondary substrates. Gemfibrozil analog and ribose analog could be prepared in 9% and 42% of RCC. Significantly, radiosynthesis of radiolabeled ribose analogs by conventional substitution reactions was limited due to the sulfonate precursor readily decomposed at room temperature (D’Angelo and Taylor, 2016).

Difluorocarbene intermediate is a powerful tool in organic synthesis (Ni and Hu, 2014). It can readily recombine fluorine anion to prepare a trifluoromethyl group *in situ* (Zheng et al., 2015a; Zheng et al., 2015b; Zheng et al., 2017). Gouverneur and co-workers reported a one-step route to [^18^F]CF_3_SO_2_NH_4_ from [^18^F]fluoride, (triphenylphosphonio)acetate (PDFA, difluorocarbene precursor) with 1,4-diazabicyclo [2.2.2]octane bis(SO_2_) adduct (DABSO) or *N*-methylmorpholine·SO_2_ (NMMSO_2_) (SO_2_ source) and it applied to selective C-H bonds [^18^F]trifluoromethylation of peptides (Kee et al., 2020) (Figure 2D1). [^18^F]Trifluoromethyl radical could smoothly generate form [^18^F]CF_3_SO_2_NH_4_ in presence of Fe(III) salts and TBHP. For unmodified peptides, direct C−H bonds [^18^F]Trifluoromethylation selective occurred at L-tryptophan or L-tyrosine. Biologically relevant peptides, including immunomodulator thymogen (oglufanide) (Deigin et al., 2007), endomorphin-1 (a tetrapeptide related to Alzheimer’s disease) (Frydman-Marom et al., 2011), somatostatin-14 (a cyclic tetradecapeptidic hormone with a broad inhibitory effect on endocrine secretion) (Vale et al., 1972), melittin (a 26-residue venom peptide) (Raghuraman and Chattopadhyay, 2007), octreotide (an octapeptide that mimics natural somatostatin) (Pauwels et al., 2019), underwent Trp-selective [^18^F]trifluoromethylation; Angiotensin fragment 1–7 (a peptide with anti-inflammatory properties) (El-Hashim et al., 2012), c (RGDyK) (a peptide ligand of integrin α_v_β_3_ receptors) (Cai et al., 2006), underwent Tyr-selective [^18^F]trifluoromethylation. It was worth noting that the C-H bonds [^18^F]trifluoromethylation of recombinant human insulin (MW = 5,808 Da) were also successful. [^18^F]CF_3_-octreotide was automated radio-synthesized with the molar activity of 0.28 GBq/μmol.

Diazo compounds are known as carbene precursors to react rapidly with transition metals to form electrophilic metal carbenoids under mild conditions (Ford et al., 2015). Doyle and co-workers developed copper-catalyzed radiofluorination of *α*-diazocarbonyl compounds in mild conditions with [^18^F]KF as a result of synthesizing *α*-[^18^F]fluorocarbonyl products with the typical molar activity of 48.1 GBq/μmol (Gray et al., 2016) (Figure 2D2). The traditional nucleophilic [^18^F]fluorination reaction conditions are not befitting for the radiosynthesis of most *α*-[^18^F]fluorocarbonyl targets (Liu et al., 2011). They showed the RCY of this radiofluorination protocol is significantly higher than in the previous literature. Some known radiotracers for positron emission tomography were readily accessible using this practical approach. *N* ^5^-[^18^F]fluoroacetylornithine (*N* ^5^-[^18^F]FAO) (Turkman et al., 2011) was labeled in 39% of RCC (only 8% of RCY by the previous S_N_2). *N*-(2-(diethylamino)ethyl)-2-[^18^F]fluoropropanamide ([^18^F]FPDA) (Liu et al., 2013) was labeled in 45% of RCC in one step (merely 3% of overall RCY with many radiochemical steps in prior method). Peptides and glycosides were also compatible with this radiofluorination.

The strategy of directly and selectively transforming C-H bonds to C-^18^F bonds is helpful due to the needlessness for the pre-functionalization of labeling precursors (Szpera et al., 2019). Groves, Hooker, and co-workers presented manganese porphyrin mediated direct radiofluorination of unactivated aliphatic C-H bonds with [^18^F]fluoride (Liu et al., 2018) (Figure 2E). Similar to the earlier mentioned protocol (Huang et al., 2015), the anion exchange cartridge was eluted by acetone/acetonitrile solution of Mn^III^(TPFPP)OTs. Amino acid transporter, such as ACPC, leucine, valine, tyrosine analogs and leucine containing dipeptide, Lyrica analogs (an anticonvulsant drug) (Dworkin and Kirkpatrick, 2005), amantadine analogs (an antiparkinson disease drug), ezetimibe (a cholesterol-lowering drug), flutamide (a prostate cancer drug) (Baker et al., 1967), were radiolabeled efficiently at the aliphatic C-H bonds. They hypothesized that ^18^F-labeled leucine and valine analogs have never been reported due to the inaccessibility of their corresponding precursors. However, C-H bonds radiofluorination occurred at the tertiary carbon atom due to the *ortho*-position alkyl group can stabilize the reaction intermediate. Herein, the labeling precursors with *gem*-dialkyl groups are required for this C-H bonds radiofluorination reaction.

## Radiosynthesis of Fluoroarenes With [^18^F]Fluorides

**FIGURE 3:**
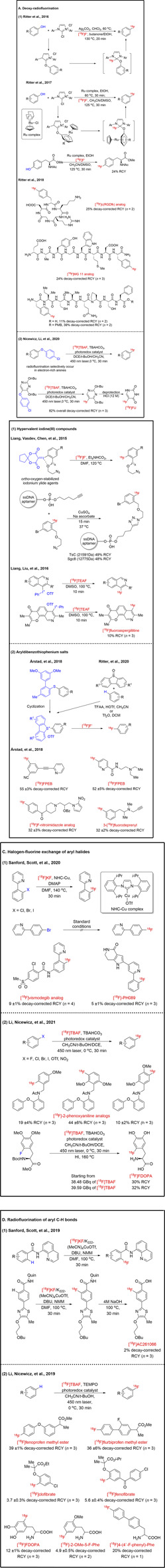
(Continued).

Aromatic nucleophilic substitution (S_N_Ar) reaction is a widely practiced method for the construction of [^18^F]fluoroarenes with [^18^F]fluoride (Preshlock et al., 2016). An activating group and a leaving group on the arene to stabilize the Meisenheimer complex are necessary for the highly efficient introduction of fluoride into fluoroarenes by S_N_Ar (Bunnett and Zahler, 1951). Despite significant advances in the ^18^F-labeling of electron-deficient arenes, there is still a huge amount of need for efficient methods for the [^18^F]fluorination of electron-neutral and electron-rich arenes (Born et al., 2017). Herein, a new mechanism avoiding Meisenheimer intermediate or novel [^18^F]fluoroarene precursors carrying new activating and leaving groups remains to be discussed. Furthermore, novel halogen-[^18^F]fluorine exchange reactions and C-H bond radiofluorination will also be discussed (Figure 3).

Phenols are frequently-used moieties in organic compounds (Qiu and Li, 2020), which makes deoxy-radiofluorination of phenols becoming an attractive strategy to achieve fluoroarenes (Tang et al., 2011). Ritter and co-workers presented a distinctive deoxy-radiofluorination method of phenols based on a concerted nucleophilic aromatic substitution (CS_N_Ar) reaction (Neumann et al., 2016) (Figure 3A1). Compared with the traditional aromatic nucleophilic substitution (S_N_Ar) mechanism (Bunnett and Zahler, 1951), CS_N_Ar does not proceed via a Meisenheimer intermediate. Herein, a wide variety of functional groups including amines, amides, thioethers, and heteroarenes were tolerated for this deoxy-radiofluorination. One year later, Ritter and co-workers further utilized a ruthenium-mediated deoxy-radiofluorination of phenols (Beyzavi et al., 2017). Compared with previous work, this ruthenium-mediated deoxy-radiofluorination reaction expanded the substrate scope to even the most electron-rich phenols. Ruthenium reduced the electron density of phenols and accelerated nucleophilic aromatic substitution of phenols. They were able to perform the reaction in a fully automated mode and get the isolated protected [^18^F]fluorophenylalanine derivative in 24% of activity yield with the molar activity of 93 GBq/μmol. Site-specific deoxy-radiofluorination of small peptides with [^18^F]fluoride also had been reported by Ritter and co-workers (Rickmeier and Ritter, 2018). Small peptides that could potentially be used as PET tracers, such as the c (RGDfk) analog (an angiogenesis monitoring PET tracer) (Cai and Conti, 2013), MG 11 analog (a gastrin-releasing peptide receptor tracer) (Good et al., 2008), were successfully labeled by this protocol. In their previous work, the substrates with C-terminal free carboxylic acid suffered from low yields during radiolabeling (Neumann et al., 2016). In this work, they showed the protection of C-terminal free carboxylic acid with *p*-methoxybenyzl (PMB) ester effectively increased the RCY. The typical peptide was automated radio-synthesized with the molar activity of 99 GBq/μmol.

Nicewicz, Li, and co-workers demonstrated another deoxy-radiofluorination mechanism called cation-radical-accelerated S_N_Ar (CRA- S_N_Ar) (Tay et al., 2020) (Figure 3A2). A novel strategy for polarity-reversed photoredox catalyzed deoxy-radiofluorination of electron-rich phenol derivatives with [^18^F]TBAF was presented. Photoredox catalyzed deoxy-radiofluorination selectively occurred in the electron-rich arenes under mild conditions with moderate-to-excellent RCYs. Highly efficient radio-synthesized 5-[^18^F]fluorouracil ([^18^F]FU), which is an antimetabolite used to treat certain cancers (Saleem et al., 2000), was produced in two steps, including deoxy-radiofluorination and deprotection with an overall 82% of decay-corrected RCY with the molar activity of 74.7 GBq/μmol. This method was supplementary to existing ways that involve hypervalent iodoniums (Rotstein et al., 2014) and aryl nickel complexes (Hoover et al., 2016).

Hypervalent iodine (III) compounds as novel activating and leaving groups play a pivotal role in nucleophilic [^18^F]fluorination of non-activated arenes (Deng et al., 2019). Pike and co-workers demonstrated the first example of radiofluorination with diaryliodonium salts, whereby both electron-deficient and electron-rich arenes showed a high ^18^F-labeling efficiency (Pike and Aighirhio, 1995). During modifying the structure of hypervalent iodine (III) compounds, Liang, Vasdev, Chen, and co-workers utilized the *ortho*-effect and developed an *ortho*-oxygen-stabilized iodonium ylide agents (Wang et al., 2015) (Figure 3B1). Compared with Pike’s work (Pike and Aighirhio, 1995), they speculated that a secondary bonding interaction between *ortho*-oxygen and hypervalent iodine would provide stabilization for iodine (III) to yield thermally stable and highly reactive. The azide moiety of ^18^F-labeled products, the molar activity greater than 74 GBq/μmol, easily underwent [3 + 2] cycloaddition or coupling with alkyne-containing small or biological molecules, such as ssDNA aptamer TsC (21591Da) and Sgc8 (12775Da) (Jacobson et al., 2015). In previous work, TsC aptamer radiolabeled in only 1.5% of RCY. The RCY raised to 49% of RCY by using this novel method. Recently, Liang, Liu, and co-workers reported a general protocol for the preparation of [^18^F]fluoroisoquinolines with radiochemical conversion up to 92% with the molar activity of 56.6 GBq/μmol (Yuan et al., 2016). As proof of concept [^18^F]fluoroaspergillitine, a fluorinated marine natural product, was prepared in 10% of isolated radiochemical yield.

Beside the diaryliodonium salts, aryldibenzothiophenium salts can be used as another catalyzer for synthesizing non-activated arene [^18^F]fluorides by nucleophilic fluorination. Årstad and co-workers demonstrated regioselectively radiofluorination of dibenzothiophene sulfonium salts (prepared by biaryl thioethers via intramolecular ring-closing reaction) with [^18^F]fluoride afford [^18^F]fluoroarenes (Gendron et al., 2018) (Figure 3B2). 3-[^18^F]fluoro-5-(pyridine-3-ylethynyl)benzonitrile ([^18^F]FPEB, a metabotropic glutamate 5 receptors tracer) (Wang et al., 2007), ^18^F-labeled nitroimidazole analog (a hypoxia tracer), *N*-[2-(diethylamino)-ethyl]-5-[^18^F]fluoropicolinamide ([^18^F]P3BZA, a melanoma tracer) (Ma et al., 2019), 3-[^18^F]fluorodeprenyl (a monoamine oxidase B tracer) (Fowler et al., 2015), were successfully radio-synthesized by this [^18^F]fluorination method. 3-[^18^F]fluorodeprenyl was radio-synthesized with the molar activity of 17–30 GBq/μmol. To achieve radiosynthesis of [^18^F]FPEB, they attempted to prepare the corresponding triarylsulfonium salt but failed. For [^18^F]P3BZA, the reported radiosynthesis is low yielding. Followed by the new [^18^F]fluorination method, the RCY raised from 12% to 52%. Ritter and co-workers reported a novel site-selective late-stage aromatic [^18^F]fluorination method via aryl sulfonium salts (Xu et al., 2020). Significantly, they showed how electronically different dibenzothiophenes appropriately matched the electronic requirements of the arene. Heterocycles, halides, amides, and sulfonamides were tolerated for this [^18^F]fluorination reaction and a range of small-molecule drugs were successfully labeled.

Aryl halides are the most wildly used radiofluorination precursors due to their characteristics of being stable and synthetically accessible (Preshlock et al., 2016). Halogen-fluorine exchange reactions are limited for radiofluorination of electron-deficient arenes via S_N_Ar (Preshlock et al., 2016). Nevertheless, it is still a huge challenge for radiofluorination of unactivated arenes via halogen-fluorine exchange. Sanford, Scott, and co-workers described the ligand-directed *N*-heterocyclic carbene (NHC) Cu complexes mediated radiofluorination of aryl halides with the typical molar activity of 1.6 GBq/μmol (Sharninghausen et al., 2020) (Figure 3C1). They showed that directing groups pattern on the *ortho*-position of halogen substituents was necessary for this reaction. These substrates with bromo-substituent on the *para*-position of directing groups did not afford desired products under standard conditions. Vismodegib analog, a basal cell carcinoma treatment drug (Dlugosz et al., 2012), and PH089, an MK-2 inhibitor (Anderson et al., 2007), smoothly underwent radiofluorination. Li, Nicewicz, and co-workers demonstrated a method for constructing aryl C-^18^F bonds through direct halogen-fluorine exchange on electron-rich arene halides under mild photoredox conditions (Chen et al., 2021) (Figure 3C2). ^18^F-labeled 2-phenoxyaniline analogs as translocator protein (TSPO)-specific PET tracers for neuroinflammation imaging have been investigated (Werry et al., 2019). Using their halogen-fluorine exchange method, the ^18^F atom was successfully introduced into potential new imaging agents targeting TSPO. This novel protocol offered an opportunity to radiosynthesis and explore a series of ^18^F-labeled *O*-methyl tyrosines as PET tracers in an MCF-7 tumor model. For clinically relevant scaling, FDA approved PET tracer [^18^F]FDOPA was obtained with >30% of RCY and molar activity of 1.5 GBq/μmol in 100 min by using this method. For this radiofluorination reaction, substrates with *O*-atom, *N*-atom, or *S*-atom at *ortho*- or *para*-position of halides were necessary.

Compare to traditional methods of radiofluorination, such as the Balz-Schiemann reaction, deoxy-fluorination, and S_N_Ar reaction, C-H bonds radiofluorination methods are favorable due to needless pre-functionalization of the substrate (Preshlock et al., 2016; Szpera et al., 2019). Sanford, Scott, and co-workers disclosed that 8-aminoquinoline directing groups enable Cu-mediated aromatic C-H bonds activation and nucleophilic radiofluorination with [^18^F]KF (Lee et al., 2019) (Figure 3D1). This aromatic C-H bonds radiofluorination method was applied to a series of biologically relevant molecules [^18^F]AC261066, a RARβ2 agonist (Lund et al., 2005), was automated radio-synthesized in two steps, radiofluorination, and hydrolysis of the directing group, with 2% of decay-corrected RCY with the molar activity of 29.6 GBq/μmol. Li, Nicewicz, and co-workers disclosed a mild condition for direct radiofluorination of aromatic C-H bonds under organic photoredox catalyzed conditions with the typical molar activity of 51.8 GBq/μmol with 2,2,6,6-tetramethylpiperidinooxy (TEMPO) as oxidant and tetrabutylammonium [^18^F]fluoride ([^18^F]TBAF) as [^18^F]fluoride source (Chen et al., 2019) (Figure 3D2). Radiofluorination mainly occurred at the *para*-position of electron-donating groups; when the *para*-position was substituted, radiofluorination occurred at the *ortho*-position of electron-donating groups. Nonsteroidal anti-inflammatory drugs (NSAIDs) are an important class of pharmaceuticals that alleviate pain and inflammation. The NSAID derivatives (Cryer and Feldman, 1998), fenoprofen methyl ester, and flurbiprofen methyl ester were radiofluorinated in 39% and 36% of decay-corrected RCYs. Restricted by the radiolabeled method, well-studied ^11^C-labeled NSAID derivatives had the disadvantage of a shorter half-life than fluorine-18. The hypolipidemic agents, clofibrate and fenofibrate, were selectively fluorinated in moderate decay-corrected RCYs. [^18^F]FDOPA, a PD and neuroendocrine tumors PET tracer (Pretze et al., 2017), was radio-synthesized in two steps with 12% of decay-corrected RCY. Extensive and sensitive [^18^F]FDOPA precursors were required in published routes. The protected *O*-Me-*ortho*-tyrosine and 4-phenyl-phenylalanine were also successfully radiofluorinated, and their deprotected forms were accessed with relative ease.

## Radiosynthesis of Fluoroalkenes With [^18^F]Fluorides

**FIGURE 4:**
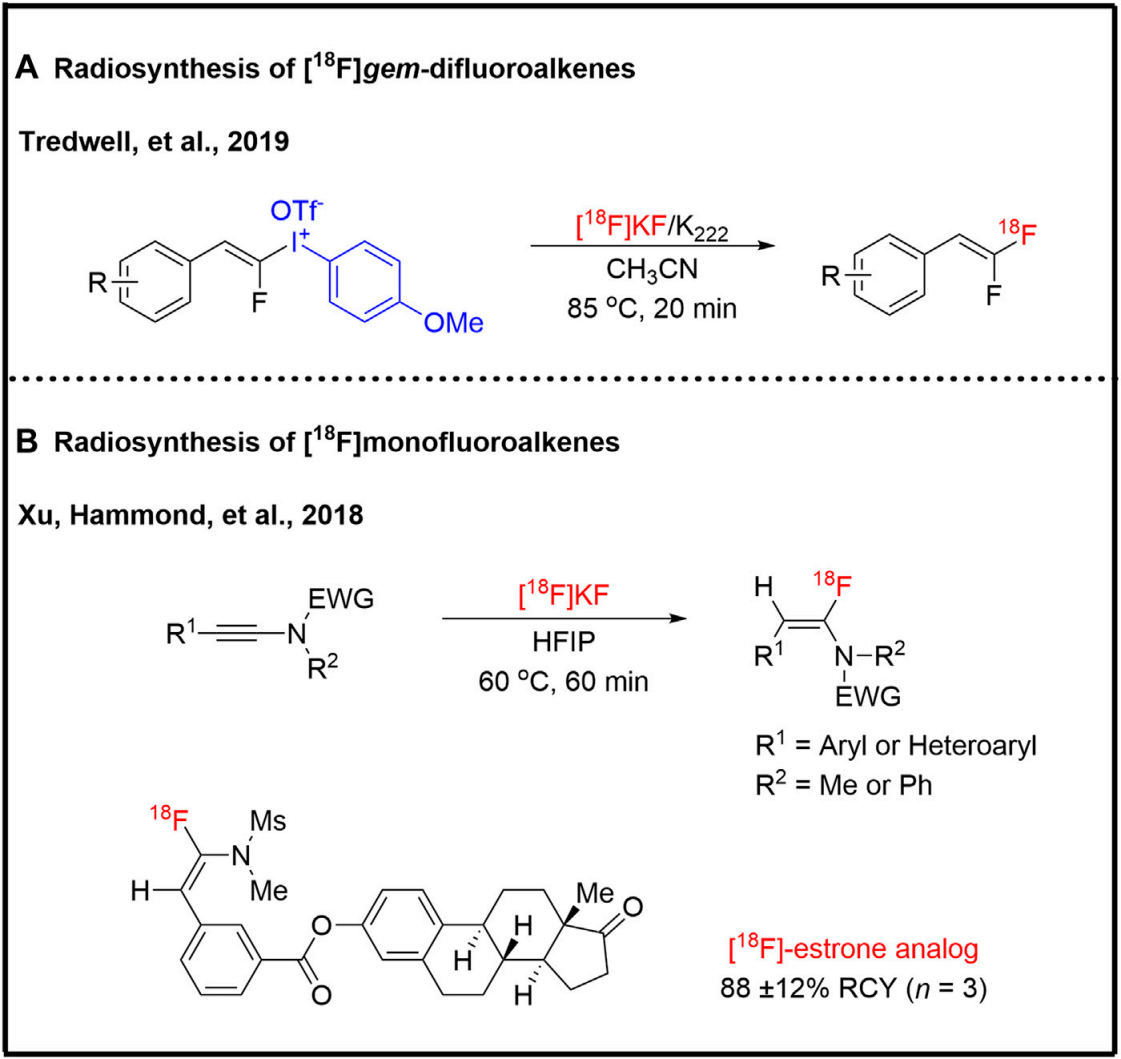
Radiosynthesis of fluoroalkenes.

*Gem*-difluoroalkene moiety presents in several drug molecules, such as numerous enzyme inhibitors, due to the similar bioisosteric to a carbonyl group (Shen et al., 2014). The [^18^F]*gem*-difluoroalkenes were obtained as byproducts in radiofluorination of corresponding difluoroalkenes via an addition-elimination mechanism (Fawaz et al., 2014). Tredwell and co-workers reported the synthesis of [^18^F]*gem*-difluoroalkenes with the typical molar activity of 1.0 GBq/μmol from [^18^F]fluoride and fluoroalkenyl (4-methoxyphenyl)iodonium triflates (Frost et al., 2019) (Figure 4A). The [^18^F]*gem*-difluoroalkenes can be easily translated into 1,1-[^18^F] difluoromethylene-containing groups. This transformation supplied another method of radiosynthesis of non-benzylic geminal [^18^F]CF_2_ groups. Monofluoroalkene moiety can be used as a peptidomimetic unit in the design of protease inhibitors as well as positron emission tomography probes based on the similar charge distribution and dipole moment between amide bond and fluoroalkene moiety (Zhang et al., 2009). Xu, Hammond, and co-workers offered a reliable protocol for the synthesis of ^18^F-labeled monofluoroalkene via hydrogen-bonding enabled radiofluorination of ynamides (Zeng et al., 2018) (Figure 4B). They demonstrated the hydrogen-bonding network generated from hydrogen-bond-donor solvents accelerates the rate-determining proton-transfer step. To demonstrate the applicability, an ^18^F-labeled biologically active estrone derivative was prepared with great efficiency (Figure 4).

## Heteroatom-^18^F Bonds Formation

**FIGURE 5:**
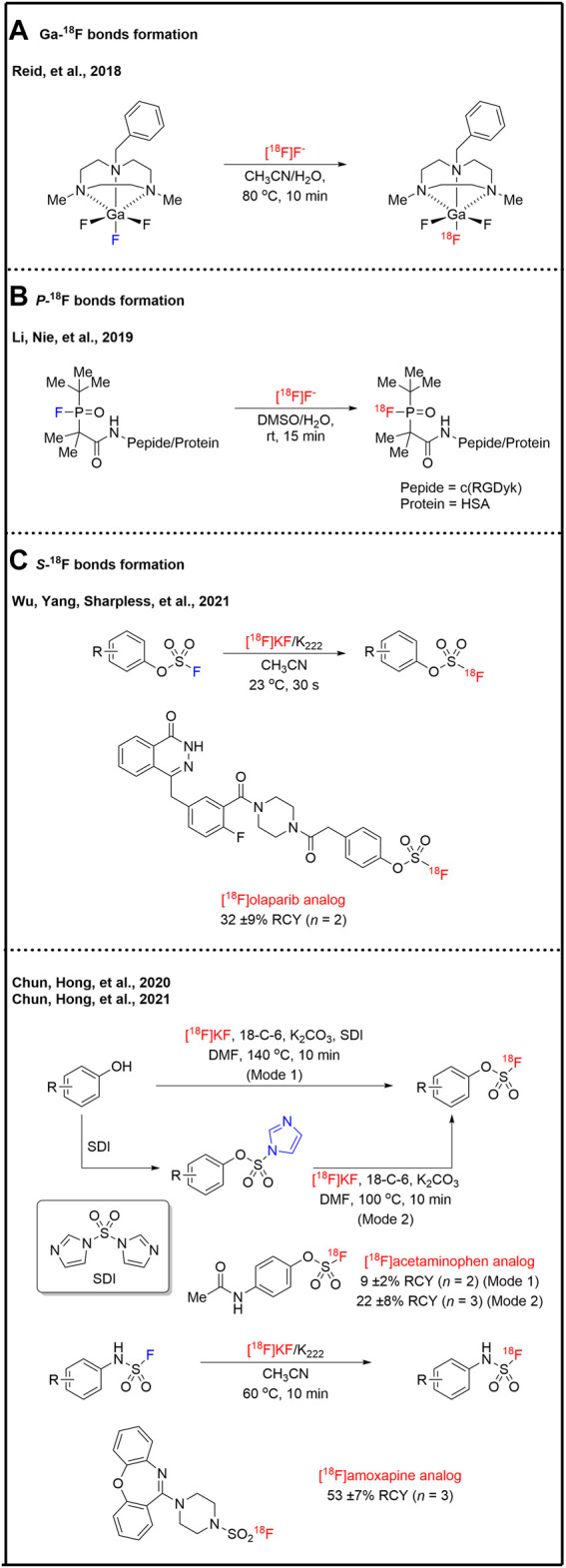
Heteroatom-^18^F bonds formation.

Expect the traditional ^18^F-labeling strategies of C-^18^F bond formation, the noncanonical strategies of hetero-^18^F bond formations, such as B-^18^F, Al-^18^F, Si-^18^F, Ga-^18^F, P-^18^F, and S-^18^F bonds, which show the unique properties in positron emission tomography probes design. B-^18^F, Al-^18^F, and Si-^18^F derivatives as PET tracers have been excellently reviewed by Gabbai and co-workers (Chansaenpak et al., 2016), and Schirrmacher, and co-workers (Wängler et al., 2012). Herein, B-^18^F, Al-^18^F, and Si-^18^F bond formation warrants a brief discussion. Within the Group 13 elements, B-^18^F derivatives are the most studied PET applications (Monzittu et al., 2018). The research on Al-^18^F provided the first example of a metal chelate system for [^18^F]fluoride capture in water (Laverman et al., 2010). However, B-^18^F, Al-^18^F, and Si-^18^F derivatives have obvious weaknesses (Hong et al., 2019), such as specific pH requirement (B-^18^F derivatives), the steric effect of bulky chelate synthons (Al-^18^F derivatives), limited stability and high lipophilicity (Si-^18^F derivatives) and potential biosafety issue due to possible metal contamination. Reid and co-workers synthesized and demonstrated 1-benzyl-4,7-dimethyl-1,4,7-triazacyclononane (BnMe_2_-tacn) liganded GaF_3_ complex is extremely stable in water (Bhalla et al., 2014). Then, they presented a simple and rapid method for ^18^F-labeling of [^18^F]GaF_3_(BnMe_2_-tacn) complex by isotopic exchange with the molar activity of 675 MBq/μmol (Monzittu et al., 2018) (Figure 5A). This ^18^F–^19^F exchange method significantly decreased the concentration of the GaF_3_(BnMe_2_-tacn) compare to the previous ^18^F-Cl exchange reaction (Bhalla et al., 2014). Organophosphine [^18^F]fluorides also had been prepared by Li, Nie, and co-workers by isotopic exchange (Hong et al., 2019). They illustrated that steric hindrance is critical for the stability of organophosphine [^18^F]fluorides (Figure 5B). Human serum albumin (HSA), a heat-sensitive globular protein, was radiolabeled at room temperature and applied to blood pool imaging with the molar activity of 1.1 GBq/μmol. Wu, Yang, Sharpless, and co-workers reported an ultrafast (within 30 s) isotopic exchange method for the radiosynthesis of aryl [^18^F]fluorosulfates (Ar-OSO_2_F) which was the first PET imaging application of S−^18^F based probes (Zheng et al., 2021) (Figure 5C). ^18^F-labeled olaparib analog was successfully radio-synthesized in 32% of RCY with the molar activity of 280 GBq/μmol. Aryl [^18^F]fluorosulfates were also successfully radio-synthesized in two modes by Chun, Hong, and co-workers (Kwon et al., 2020). The radiofluorination modes include the direct one-pot radiofluorosulfurylation of phenolic precursors (Mode 1) and the radiofluorination of isolated imidazylates (Mode 2). The radiofluorination of isolated imidazylates (Mode 2) showed higher RCYs. Both Mode 1 and 2 afforded similar molar activity. ^18^F-labeled acetaminophen analog was automated radio-synthesized in 9% of decay-corrected RCY with the molar activity of 42 GBq/μmol (Mode 1) and 22% of decay-corrected RCY with the molar activity of 55 GBq/μmol (Mode 2). Based on previous works, Chun, Hong, and co-workers radio-synthesized sulfamoyl [^18^F]fluorides by the ^18^F–^19^F exchange method (Jeon et al., 2021). ^18^F-labeled amoxapine derivative of sulfamoyl fluoride was automated radio-synthesized in 53% of RCY with 56 GBq/μmol (Figure 5).

## Summary

Recently, the interest in the development of novel ^18^F-labeled PET tracers increased rapidly. New methods, new reagents, and new structures have been investigated for synthesizing [^18^F]fluoroalkanes [^18^F]fluoroarenes [^18^F]fluoroalkenes, and [^18^F]fluorine-heteroatom containing compounds. Commercially available and inexpensive labeling precursors are beneficial for PET tracer design. Alcohols, phenols, and carboxylic acid are frequently-used moiety in natural products and pharmaceutical molecules. For this reason, deoxy-radiofluorination, decarboxylative radiofluorination, and C-H bonds radiofluorination have the advantage. Among them, C-H bonds radiofluorination has the greatest advantage, but it also has the disadvantage of poor regioselectivity. Halogen-[^18^F]fluorine exchange and [^18^F]fluorination of difluorocarbene provide reliable methods of access to [^18^F]fluoroalkyl groups, such as -[^18^F]SCF_3_, -[^18^F]CF_3_, et al. Innovation in reagents and structures is led by the new radiosynthesis methods. The new reagents [^18^F]Umemoto reagent and [^18^F]CF_3_SO_2_NH_4_, allow the introduction of [^18^F]trifluoromethyl into bioactive molecules and biologically relevant peptides. The new structures, [^18^F]fluoroalkenes, and novel [^18^F] fluorine-heteroatom-containing compounds have been successfully synthesized. It should also be noted that the research of PET tracer based on [^18^F]fluoroalkenes is rare. The application of these novel protocols accelerates the progress of PET tracer design and allows PET tracers to synthesize on a clinically relevant scale.
